# Cognition, sleep and estrogen—menopause is really not just about hot flashes

**DOI:** 10.1111/aogs.14471

**Published:** 2022-10-20

**Authors:** Annika Auranen

**Affiliations:** ^1^ Department of Obstetrics and Gynecology, Tays Cancer Center Tampere University Hospital Tampere Finland

Menopausal transition is often presented, in particular in lay publicity, as a period of embarrassing hot flashes and irritable mood, features that are easily subject to comical images and comments. There is nothing embarrassing about going through natural phases of ones biological life. The importance of menopause as one of the significant milestones and turning points in a woman's biological life is maybe not completely understood. The incidence of many significant chronic diseases, such as cardiovascular diseases and osteoporosis starts to increase 10–15 years after menopause, and the time window just after menopause can give an opportunity for preventive measures against these later appearing conditions.

A recently published review by Jett et al. summarizes what is known about estrogen's role in the female brain.^1^ Estradiol receptors are present in the brain, and estradiol can also be locally synthetized in the brain. These two types of estradiol, produced by the ovaries and by the brain, control various neurobiological processes including regulation of body temperature, response to stress and some aspects of mood and cognition. The function of estradiol and estrogen receptors is neuroprotective. Changes in estradiol concentrations appear to have effect on cognitive function. In the Study of Women Across the Nation (SWAN), over 2300 women were followed for over 4 years. A decrease in verbal memory and processing speed in perimenopause was noted, but these changes returned to normal or nearly baseline levels after menopause.^2^ Similar findings have been reported also in other studies.

The incidence of the most common form of dementia, Alzheimer's disease is rising as the population grows older. In the US, 12% of women over 65 and 9% of men over 65 have Alzheimers disease. The disease appears to be more common in women, and it is possible, that there are gender differences (hormonal, societal, chromosomal) on reasons for developing Alzheimer's disease.^3^ The objective findings in Alzheimer's disease are the accumulation of beta‐amyloid plaques outside neurons and the accumulation of abnormal form of the protein tau inside neurons.

The discovery of the glymphatic system, a “pseudo‐lymphatic” perivascular network that is capable of clearing brain metabologic waste, has highlighted the role of good sleep in brain health.^4^, ^5^ The glymphatic system is active in sleep, and the wast majority of glymphatic clearance occurs in sleep and in particular, slow‐wave sleep. The percentage of slow‐wave sleep is highest during puberty, after which it gradually starts to decline. Short sleep duration is associated with greater beta amyloid burden in the brain of elderly individuals. It appears, that if a person suffers from poor sleep quality, meaning fragmented or short sleeping periods, the “washing machinery” of the brain does not function properly, allowing the harmful proteins to accumulate in the brain.

The finding that good sleep perhaps protects from Alzheimer's disease has relevance to estrogens and menopause. Sleeping disorders are common in menopausal transition and also in the postmenopausal period. Typically, the sleeping disorder manifests itself as waking up too early (short sleep) and fragmented sleep. Recently, results from a British cohort study, including nearly 8000 women with a 25 year‐follow‐up were published. In this study, sleep parameters were evaluated with repeated questionnaires, and part of the participants also participated in an accelemetor substudy, in which the participants used a wrist‐worn accelemetor to register sleep duration. Dementia diagnosis were acquired via linkage to health registries. The study showed, that short sleep duration in midlife is associated with an increased risk of late‐onset dementia.^6^

The health effects of hormone replacement therapy (HRT) have been studied in many observational and also randomized and case–control studies during the last decades. Although these studies often have not been absolutely perfectly designed, we can be quite confident, that HRT prevents osteoporosis, can protect from cardiovascular disease if started early in menopause, and can increase breast cancer risk, if estrogen‐progesterone therapy is given. However, our understanding of estrogens' effects on cognitive functions and maybe prevention of dementia is very limited at the moment.

The societal impact of cognitive disorders and dementia and also the individual impact on one's wellbeing and quality of life is becoming more and more important as the aging population grows. Therefore, it is time to actively think about preventive measures for these conditions.^7^ Time and continuing research will eventually tell us whether estrogen could be used for prevention on dementia and cognitive deterioration. Until then, the best approach is to promote a lifestyle that also benefits the brain: good sleep, sensible alcohol comsumption and no smoking.
